# Determinants of p53 DNA binding, gene regulation, and cell fate decisions

**DOI:** 10.1038/s41418-024-01326-1

**Published:** 2024-06-29

**Authors:** Martin Fischer, Morgan A. Sammons

**Affiliations:** 1 Computational Biology Group, Leibniz Institute on Aging – Fritz Lipmann Institute (FLI), Beutenbergstraße 11, 07745 Jena, Germany; 2 Department of Biological Sciences and The RNA Institute, The State University of New York at Albany, 1400 Washington Avenue, Albany, NY 12222 USA

**Keywords:** Tumour-suppressor proteins, Gene regulation

## Abstract

The extent to which transcription factors read and respond to specific information content within short DNA sequences remains an important question that the tumor suppressor p53 is helping us answer. We discuss recent insights into how local information content at p53 binding sites might control modes of p53 target gene activation and cell fate decisions. Significant prior work has yielded data supporting two potential models of how p53 determines cell fate through its target genes: a selective target gene binding and activation model and a p53 level threshold model. Both of these models largely revolve around an analogy of whether p53 is acting in a “smart” or “dumb” manner. Here, we synthesize recent and past studies on p53 decoding of DNA sequence, chromatin context, and cellular signaling cascades to elicit variable cell fates critical in human development, homeostasis, and disease.

## Facts

- Transcription factors contain DNA binding domains that enable them to recognize and bind short DNA sequences.
- Sequence content and context in gene regulatory elements, and how that influences transcription factor occupancy, plays a crucial role in development and disease.
- p53 is a model transcription factor, due in part to decades of investigation in the context of tumor suppression and cancer and more recently as a critical barrier to successful gene editing approaches.
- Similar to other transcription factors, p53 binding to the genome is mediated by its DNA recognition motif, the p53 response element (p53RE), and is affected by DNA shape, chromatin state, and co-factors [1, 2].
- p53 controls cell fate after DNA damage and other cellular insults, such as the decision to undergo apoptosis or to temporarily or permanently arrest the cell cycle, through its ability to regulate gene expression.

## Open Questions

- How do transcription factors like p53 “choose” which genomic binding sites to occupy, which genes to regulate, and ultimately, what cell fate is the best outcome at the cellular and organismal level?
- How do DNA sequence, DNA shape, chromatin structure, p53 binding affinity and kinetics, and other non-DNA information like cell and tissue context affect p53-dependent transcription and cell fate?
- How do other transcription factors and co-factors support or impede p53 activities on DNA and what influence do these factors have on tumor suppression and other critical p53-dependent activities?
- How can we best modulate a cell’s apoptotic threshold to make p53 activating cancer treatment strategies more successful?

## Introduction

### Cell fate decisions by “smart” and “dumb” p53

**Fig. 1:**
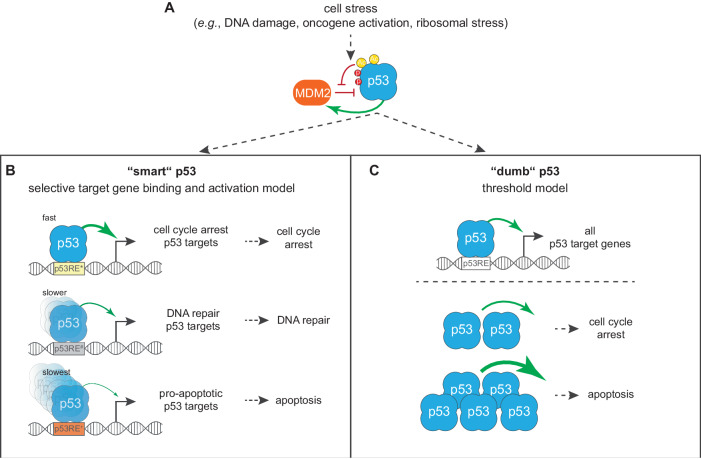
Two models for p53-dependent cell fate control. **A** p53 is kept inactive via MDM2-mediated E3 ligase activity, which itself is inhibited by stress-dependent post-translational modifications (PTMs) to p53. In the absence of MDM2-mediated degradation, p53 protein levels rise and p53 activates transcription of a broad gene network and dictates cell fate. **B** The “smart” model suggests that differential binding kinetics of p53 to target gene regulatory sequences dictates cell fate decisions by p53. In this model, intrinsic sequence characteristics (DNA sequence, shape) or modulation of p53 protein activity (PTMs, co-factor binding) drive p53-mediated transcription of selective genes controlling distinct cell fates. **C** The “dumb” model suggests that p53 protein abundance dictates p53-dependent cell fates. In this model, p53 binds to all permissive p53REs and broadly activates all target genes. As p53 protein levels rise, interactions between p53 and cell type and condition-dependent factors ultimately determine cell fates.

The tumor suppressor p53 is best known as a transcription factor that uses its target genes to control cell fate decisions in response to cellular stress [3]. The regulation of p53 revolves around the E3-ubiquitinase MDM2, which binds p53 and leads to its proteasomal degradation in unstressed cells. Upon stress, p53 is post-translationally modified to block its interaction with MDM2, leading to elevated p53 levels [4] (Fig. 1A). Downstream of the numerous pathways that can be driven by p53, cell cycle arrest and apoptosis are arguably the most prominent fates a cell can arrive at. Thus, p53 can control the fate of a cell to survive or die in response to stress. Given the critical importance of cellular life-or-death decisions to anti-cancer strategies, therapeutic genome editing approaches, and our expanding view of aberrant p53 activity in developmental disorders, a large number of studies have addressed the question of how the p53 signaling pathway makes this ultimate decision about a cell’s life or death [5]. Similar to most of these studies, this Review focuses on the role of full-length p53 in mammals. Many p53 target genes have been associated with different pathways and cell fates [6], such as p21 (also known as CDKN1A), which promotes cell cycle arrest [7], and puma (BBC3), which stimulates apoptosis [8, 9]. The specific associations between target genes and cell fates prompted Karen Vousden, in a review article published in 2000, to ask whether p53 is “smart” and can selectively activate specific target genes to achieve a desired cell fate, or whether p53 is rather “dumb” and tries to activate all of its target genes, leaving the ultimate cell fate decisions to other signaling cues [10]. The distinction between “smart” and “dumb” p53 serves as an analogy for our understanding of p53 functionality.

#### “smart” p53

For many years, p53 seemed rather “smart” to most researchers in the field. For example, post-translational modifications (PTMs) [11–13] and co-factors [14, 15] were identified that could help p53 to select specific target genes and ultimately tip the balance of p53-dependent cell fate decisions from survival to death and vice versa. A particularly intriguing concept, at least from a genomic perspective, suggests that differences in p53 response elements (p53REs) result in differential DNA binding affinity of p53 and varying activation kinetics of the associated target genes [16], which may contribute to p53 favoring target genes linked to cell cycle arrest over those linked to apoptosis [17, 18] (Fig. 1B). Taken together, it is thought that “smart” p53 decodes different signals that a cell generates in response to various stress conditions, and then p53 scans the genome for DNA recognition sites that encode for specific target gene activation and cell fates.

#### “dumb” p53

What if p53 was “dumb”? The strength of the p53RE, *i.e*., the predicted affinity for p53 binding, has been found to have very limited correlation with biological function [19]. Consistent with these data, p53 binds to most of its canonical target genes relatively invariant across cell types and tissues [20–23]. Furthermore, p53 activates most of its target genes at the same time, and cells undergo apoptosis when a cell type- and condition-dependent threshold of p53 levels is reached [24, 25] (Fig. 1C). In summary, “dumb” p53 welcomes any activation signal that increases its abundance and subsequently binds to any *accessible* p53RE, where it attempts to recruit Pol II to induce transcription.

The terms “smart” and “dumb” succinctly describe the information content that is present in various upstream signaling cues as well as at different levels of the p53 signaling cascade. In the following sections, we will explore our current understanding of whether p53 behaves “smart” or “dumb”, with an initial focus on target recognition and ending with cell fate decisions.

### Information content of p53REs

Through its DNA binding domain, p53 binds a DNA sequence motif known as the p53RE. The canonical p53RE is composed of two decameric half-sites with the consensus sequence RRRCWWGYYY (R = A/G, W = A/T, Y = C/T). Occasionally, multiple p53REs exist in the same regulatory element such as in *MDM2* [26] or there exist multiple regulatory elements containing a p53RE such as in *CDKN1A* [27] and *GDF15* [28]. Both of these arrangements may lead to combinatorial activity of p53 and higher target gene expression. The specific p53RE DNA sequence is variable and thus provides a means to encode critical information. This information is critical in enabling p53 to act “smart”. The information content of p53REs has been central to several models of p53 function. For example, it had been suggested that sequence differences in p53REs might determine whether p53 induces or represses transcription at a given locus [29, 30], but these models were later shown to be inconsistent with genome-wide data demonstrating that p53 is only associated with transcriptional activation at its binding sites [6, 31–35]. In general, the mechanisms that lead to the activation or repression of many genes upon p53 activation still require more in-depth study.

To make complex cell fate decisions through differential activation of target genes, the DNA sequence of a p53RE must encode information about its associated gene that p53 can effectively decode (Fig. 1B). Indeed, an early model proposed that p53REs direct p53 to regulate specific targets, such as cell cycle arrest or pro-apoptotic targets, through sequence-specific binding affinities and subsequent activation kinetics [16–18, 36]. Large-scale binding data from ChIP-seq experiments show a positive correlation between the occurrence of p53 binding and a p53RE’s similarity to the consensus sequence [37], which provides evidence that the DNA sequence of a p53RE can indeed influence p53 binding. Consistent with these data, machine learning models of DNA sequence features predict that p53-mediated activation is best described by its similarity to the consensus sequence of the p53RE [38, 39]. However, higher affinity binding sites do not always predict higher transcriptional output [40]. An early genome-wide analysis of p53RE DNA sequences and their predicted affinity for p53 binding revealed only a very limited correlation with the biological pathways of the associated genes [19]. However, a number of recent studies have provided new evidence that p53REs may encode high information content that can help p53 make decisions. Based on an evaluation of 250 p53REs, the authors of one study found that the variable DNA shape caused by the specific DNA sequence within p53REs may allow p53 to interpret directionality [41], *i.e*., p53 decodes whether to send Pol II complexes up- or downstream of the p53RE. Two other studies revived the early model in which p53REs instruct p53 to regulate cell cycle arrest or pro-apoptotic targets. While Qian et al. [17] did not identify the specific DNA information that causes p53 to favor cell cycle-associated p53REs over apoptosis-associated p53REs, the two recent studies differ markedly in the specific DNA features they identified that cause the different activation kinetics. One study reported that specific base-pair changes cause differences in the width of the minor or major DNA groove, with narrower minor grooves resulting in higher p53 binding affinity and faster target gene activation [42]. The second study found that the p53RE sequence affects DNA torsional flexibility, with high flexibility allowing for more robust p53 binding and thus faster target gene activation [43]. However, another recent study demonstrated that the DNA shape of p53REs does not generally predict its activity [39]. Although the authors have identified different mechanisms of action, they support a model in which p53 acts “smart” by decoding information from p53REs to differentially activate target genes associated with different biological functions.

Many genomic DNA fragments with p53REs have been evaluated using MPRAs (massively parallel reporter assays). Studies have used DNA fragments that bind p53 [28, 38, 44], have enhancer potential [45], or that represent essentially the entire human genome [46] and all have found that p53REs mediate significant transactivation potential. Based on these data, the authors proposed a model in which p53 binding can be sufficient to drive transcription [38, 46], implying a “dumb” p53 that attempts to activate each locus to which it binds.

In summary, the current literature contains conflicting results regarding how p53 reads and interacts with p53REs and how this translates into target gene activation. Notably, most of these observations have been made using reporter assays and do not fully recapitulate the in vivo context. These assays measure activity driven by a p53RE lacking genomic context, such as gene distance, other regulatory elements, and chromatin state. In the next section, we discuss the extent to which these properties may influence how p53 interacts with p53REs to regulate associated target genes.

### Chromatin structure affects p53 binding and productivity

**Fig. 2:**
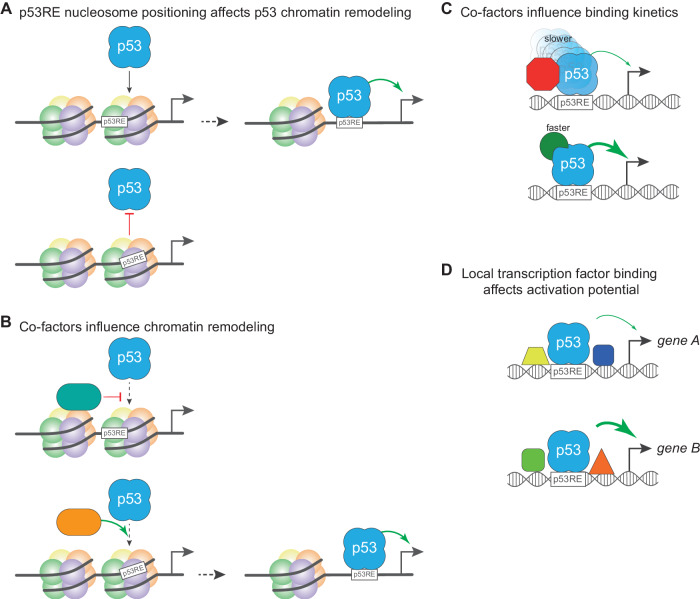
Factors modulating p53 engagement with the genome and activation of target genes. **A** p53 recognizes and binds to its RE in naked DNA and when found in certain rotational positions within nucleosomal DNA, leading to p53-dependent nucleosome remodeling and gene activation (top panel). The presence of a p53RE in other nucleosome contexts is non-permissive and prevents p53 binding and downstream gene activation (bottom panel). **B** Co-factors and chromatin remodelers can alter the local chromatin landscape to facilitate or impede p53 binding to its response element. Factors, like TRIM24, inhibit p53 activity (top panel), whereas other factors, like ATRX:DAXX and p63, promote p53 binding through nucleosome displacement (bottom panel). **C** Numerous p53 interacting proteins, *e.g*., p300 or iASPP, can alter p53:DNA binding kinetics and promote or repress p53-dependent transcription. **D** Co-binding of other transcription factors, *e.g*., SP1 or ATF3, to promoters and enhancers alters p53-dependent transcriptional activity.

Chromatin, transcription, and cell fate are inextricably linked. Chromatin structure can facilitate or impede biochemical processes on DNA depending on the context, with nucleosomes generally acting as a strong barrier to transcription factor binding [47]. Gene regulatory regions occupied by nucleosomes tend to be inactive due to this inability of transcription factors to recognize and bind their cognate DNA elements. p53 is more complicated, however, as it can recognize the p53RE in both naked DNA and in certain nucleosomal contexts as part of its pioneer transcription factor activity. When p53 interacts with nucleosome-bound p53REs, it prefers p53REs oriented near nucleosome entry/exit sites [48–51]. Conversely, p53RE positions near the nucleosome dyad, *i.e*., the center, strongly inhibit p53 binding, providing a clear mechanism to control p53 binding and activity [48–51] (Fig. 2A). Nucleosome rotational position preferences have also been proposed to differentially regulate the activation and kinetics of cell cycle control genes such as *CDKN1A* and apoptotic genes such as *BBC3/puma* [52, 53], but this rotational positioning model has not yet been tested on a high-throughput data basis.

Chromatin states may also explain observations of p53 binding and perhaps, differences in cell fate choices. The number of predicted p53 response elements (p53RE) in the human genome greatly exceeds the number of experimentally observed binding events, suggesting that DNA sequence alone is not sufficient to direct p53 binding and activity [2]. The majority of unbound p53RE are nucleosome-occupied, although the field currently lacks sufficient experimental depth and detail to determine whether these p53RE are in unfavorable rotational nucleosome positions or other restrictive chromatin states. Extensive meta-analyses of p53 genomic binding have identified two groups of p53 binding with potentially distinct activities: one invariant across cell types and another with strong cell type dependence. p53 appears to activate a common set of gene targets across cell types [21, 23, 32, 34, 54], but also activates numerous cell type-dependent targets [23, 55]. Cell type-dependent p53 binding strongly correlates with cell type-dependent differences in chromatin accessibility [22, 56], but the extent to which cell type-dependent p53 binding is causal for the observed differential activation of target genes is unclear.

Pioneer factors such as p53 recognize and bind to their DNA motifs embedded in nucleosomes in certain contexts, but also facilitate chromatin remodeling and nucleosome displacement. When does p53 binding lead to chromatin remodeling? As is often the case with chromatin structure, the answer depends on the context. In human fibroblasts, there is little evidence for p53-dependent chromatin remodeling 6 h after p53 stabilization with Nutlin-3a [57], but after 12 h of treatment with the DNA-damaging chemotherapeutic doxorubicin, p53 facilitates remodeling at a select, but still limited, number of binding sites [44]. In mouse embryonic stem cells (mESCs), p53 also led to increased chromatin accessibility after 4 h of doxorubicin treatment [58]. In addition to local chromatin regulation, p53 facilitates long-distance chromatin interactions between distal enhancers and promoters to control gene expression [59]. Still, many p53 binding locations remain “closed”, raising the question of the contexts in which p53 might remodel the local chromatin structure. Recent work in mouse embryonic stem cells provides at least one answer, but also raises additional questions. TRIM24 binds along with p53 to sites with nucleosomes containing unmethylated lysine 4 of histone H3 (H3K4). TRIM24:p53 co-binding restricts p53-dependent chromatin remodeling [58]. Conversely, transcription-associated methylation of H3K4 inhibits TRIM24 activity, thus permitting p53-dependent chromatin remodeling and activation of downstream gene targets. p53 binds to regions independent of TRIM24 that can be variably remodeled, suggesting that additional, as-of-yet unknown, cofactors also mediate p53-dependent chromatin remodeling. One such factor is the H3 chaperone ATRX:DAXX and its modulation of histone occupancy, which facilitates differential p53 binding and chromatin remodeling [60] (Fig. 2B). The breadth and impact of p53 pioneer activity, from differential binding to differential remodeling activity and target gene induction, is only beginning to be elucidated.

Although we still lack sufficiently detailed genome-wide analyses of how p53 reads and interacts with the chromatin, the current data provide the first insights into the extent to which p53 is “smart” and decodes the information itself, or whether it is “dumb” and just tries to perform its *trans*-activator function whenever possible. While the current data could be read to suggest that p53 is quite “smart” and can discriminate and bind p53REs in multiple nucleosomal contexts, the data may actually show that the chromatin structure, and in particular nucleosome positioning, can control p53’s ability to bind to and *trans*-activate a given genomic locus. In these cases, p53 does not appear to read or decode the chromatin state, but its “dumb” action is controlled by other factors that read or organize chromatin. For many sites, however, it remains unclear how p53 binding and productivity are regulated, leaving room for “smart” actions by p53.

In general, the productivity of p53 binding sites in the genome is influenced not only by the individual p53RE DNA sequence and chromatin state, but also by other factors that are located nearby. In the next section, we discuss links between such co-factors and their target-specific effects in the p53 transcriptional program.

### Co-factors influence productivity and kinetics at p53 binding sites

Multiple co-factors and other transcription factors influence p53’s ability to activate transcription at a given locus. In the previous section, we discussed how TRIM24 and ATRX:DAXX can affect p53’s ability to increase DNA accessibility and to activate transcription (Fig. 2B). Many additional factors have been identified that locally affect p53 function (Fig. 2C), including traditional co-activators like p300, which directly modifies p53 and the local chromatin environment to regulate p53-dependent transcription [61]. The ASPP (ankyrin-repeat, SH3-domain, and proline-rich-region-containing protein) family consists of three members, ASPP1, ASPP2, and iASPP. ASPP1 and ASPP2 have been shown to enhance p53 binding to promoters and activation of target genes involved in apoptosis [14], while iASPP has the opposite effect, inhibiting this process [62]. While it is unclear how ASPP1 and ASPP2 direct p53 towards target genes involved in apoptosis, iASPP has been shown to bind to p53 and perturb its interaction with p53REs, resulting in reduced binding affinity and altered selectivity [63]. Another co-factor, DAZAP2, binds to a subset of p53 targets and inhibits p53 at these sites, leading to reduced activation of the targets by p53, but it is unclear how DAZAP2 selects targets [64]. Non-protein cofactors, such as long non-coding RNAs, have a role up and downstream of p53 [65], but how they may affect local p53 activity remains largely unexplored. Interestingly, it has been shown that differences in the local assembly of the preinitiation complexes that position Pol II to transcribe the gene have been shown to be caused by different core promoter arrangements that cause p53 to rapidly induce *CDKN1A* (*p21*) of short duration and slowly induce *FAS* of long duration [66]. Such differences could contribute to differences in the magnitude and kinetics of p53 target gene activation and ultimately influence cell fate decisions.

Transcription factors have also been shown to cooperate with p53 locally (Fig. 2D). For example, SP1 and ATF3 have been shown to cooperate with p53 in target gene activation when their DNA binding sites are in close proximity [28]. A particularly critical regulator of p53 responses is its sibling p63 (ΔNp63), which is predominantly expressed in basal epithelia. p63 can bind to most sites that p53 can bind to [37], and it enables p53 to bind to specific sites that would otherwise be inaccessible to p53 [56, 67] (Fig. 2B). Combinatorial activity of transcription factors at regulatory elements is a well-studied phenomenon. This concept is comparatively understudied in the p53 network, with opposing views on the influence of local transcription factors in driving p53-dependent activities on DNA [27, 28, 38, 56]. Recent studies combining high-throughput genomic screening and modern computational approaches like deep and machine learning confirmed a long held truth about p53: regulatory elements containing a p53RE, and thus exhibiting binding of p53, drive high levels of transcription [39, 46]. While the specific roles for other transcription factors and co-factors influencing p53RE productivity were not specifically investigated, this blend of computational and experimental power will no doubt provide new insight into p53-dependent cell fate decisions.

To summarize, p53 binding and the ability of p53 to transactivate a given locus depend on multiple layers of information, ranging from the DNA sequence of the p53RE, to the nucleosomal context in which the p53RE is located, to other factors that function at the given locus. All of these properties can influence the transcriptional program of p53. They could help a “smart” p53 to decide on target gene activation and cell fate, or they could instruct a “dumb” p53 towards preferred outcomes. In the last section, we will discuss our current understanding of the extent to which the p53 transcriptional program is affected on a genome-wide scale and how this translates into different cell fate decisions.

### Gene regulation and cell fate decisions by p53

The concept that p53 can differentially activate target genes involved in different biological processes, such as cell cycle arrest and apoptosis, to achieve desired cell fates is fascinating. It is largely based on relatively low throughput analyses that have examined only a limited number of p53 target genes in cell populations. Fortunately, multiple high throughput and single cell analyses have become available and we can take an unbiased look at whether the magnitude or kinetics of p53 target gene induction varies between different modes of p53 activation and treatment durations, and how that relates to the subsequent fate of the cells.

A number of studies have evaluated the differential kinetics of p53 target gene activation. In single cells, p53 protein levels have been shown to rise in pulses in response to DNA damage, an effect that cannot be observed when cell populations are evaluated. These rapid changes in p53 protein levels have been shown to be largely driven by negative feedback regulation of p53 by MDM2 and the PPM1D phosphatase [68]. The different dynamics of p53 protein levels have been associated with different cell fates. For example, γ-irradiation resulted in p53 pulses and cell cycle arrest, and when these pulses were sustained by pharmacological treatment with the MDM2 inhibitor Nutlin-3, cells underwent apoptosis [69]. Importantly, the different p53 dynamics and cell fates were associated with different p53 target gene expression. Pulsed and sustained p53 led to the induction of cell cycle arrest genes, such as *CDKN1A*, *GADD45A*, and *XPC*, and negative feedback regulators, such as *MDM2* and *PPM1D*, but sustained p53 alone led to the activation of inducers of apoptosis, such as *APAF1*, *TP53AIP1*, and *BAX*, and senescence, such as *PML* and *YPEL3* [69]. Similar results were obtained with different doses and durations of treatment with the DNA-damaging chemotherapeutic agent doxorubicin [70]. Follow-up studies evaluated a much larger number of p53 target genes and used time-series gene expression analysis to determine the effect of p53 levels that vary over time, *i.e*., p53 pulses. These studies showed that p53 indiscriminately binds and activates its target genes in response to DNA damage, without enrichment for specific biological functions, and that subsequent differences in temporal RNA levels can be explained by differences in RNA degradation rates [20, 25, 71]. This is supported by previous data from a mouse model in which the p53-MDM2 feedback loop was disturbed by removing the p53REs from the *Mdm2* gene, resulting in increased p53 activity in response to DNA damage and induction of apoptosis, while simultaneously increasing the expression of both apoptotic and cell cycle arrest-associated p53 target genes [72]. Although there are clear differences in how p53 activates individual promoters [73–75], genes with such promoter features do not appear to be enriched for specific biological functions. This notion is also supported by earlier transcriptome analyses that failed to identify features that discriminate between p53 target genes involved in cell cycle arrest and apoptosis [24, 33] and instead suggested a rather simple model for cell fate decisions that is based on thresholds of p53 levels [24] (Fig. 1C). Consistent with p53-dependent transcript levels, low- and high-throughput chromatin immunoprecipitation (ChIP) data show that the p53 binding signal varies between promoters with p53REs but this difference does not enrich for target genes involved in specific biological functions [20, 24, 76].

In summary, the well-characterized biophysical differences in how p53 interacts with different p53REs in different nucleosomal contexts do not appear to translate into a selective mode of target gene binding or activation by p53 that has a measurable effect on cell fate. Differences in promoter properties are clearly important for the regulation of individual promoters and their associated genes, but current data suggest that this is not overly critical for cell fate decisions after p53 induction.

**Fig. 3:**
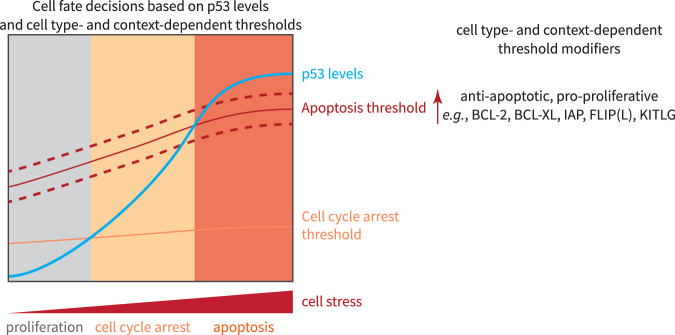
Cell fate decisions based on p53 concentration and cell type- and context-dependent thresholds. Under low stress conditions, p53 protein expression is below the threshold for cell cycle arrest or apoptosis, allowing cells to proliferate. Cell cycle arrest occurs when p53 levels reach a specific threshold, defined by a tug-of-war between pro- and anti-proliferative factors, the levels of which depend on context such as cell type and nutrient availability. When p53 levels are high enough, cells undergo p53-dependent apoptosis. The threshold for apoptosis is determined by a tug-of-war between pro- and anti-apoptotic factors that vary in their abundance depending on the cellular context. Importantly, these factors represent therapeutically actionable opportunities to tip the balance toward apoptosis, a desired outcome in cancer treatment regimens.

Other factors, such as death receptor signaling [77] and levels of the transcription factors E2F1 [78] and MYC [79], have also been shown to have threshold mechanisms that determine whether or not apoptosis is induced. A cell’s decision to induce apoptosis is largely determined by the balance of pro- and anti-apoptotic factors, the levels of which vary between cell types and contexts. Such balances provide actionable opportunities for therapeutic intervention to achieve more successful cancer treatment strategies. Differences in cells, such as cell type and context, cause different anti-apoptotic factors to be present at levels close to a tipping point toward induction of apoptosis and thus such cells may be more or less sensitive to specific interventions (Fig. 3). Over the years, several studies have shown that changes in anti-apoptotic factors can tip a cell toward apoptosis after p53 activation. Examples include BCL-2/XL [80, 81], the inhibitors of apoptosis (IAP) family [82], and the pseudo-caspase FLIP(L) [83]. Indeed, inhibition of BCL-2 and BCL-XL has been shown to be a potent anticancer strategy when combined with p53 activation [84, 85]. Another therapeutically promising example is the pathway of the integrated stress response. The gene regulatory network of ATF4, a transcription factor that is the key mediator of the integrated stress response, overlaps with the gene regulatory network of p53, specifically sharing pro-apoptotic targets [86]. Recently, PPM1D inhibition has been shown to induce ATF4 accumulation and activation in addition to stabilizing p53. Importantly, a combination treatment with PPM1D and MDM2 inhibitors resulted in a potent induction of apoptosis [87], presumably due to increased expression of pro-apoptotic target genes.

Notably, the levels of anti-apoptotic proteins can be increased in response to p53 over time, thereby raising the threshold for apoptosis induction and making the rate of p53 accumulation relevant to cell fate [82, 88] (Fig. 3). p53 leads to a cell cycle arrest fate when oncogenes are hyperactive in normal human fibroblasts or when HCT116 colon cancer cells are exposed to genotoxic damage [27, 89]. When one of the p53REs of *CDKN1A* was removed, cells failed to arrest and continued to proliferate, showing that the p53 levels were insufficient to induce apoptosis in this context [27]. In HCT116 cells, knockout of *CDKN1A* changed the cellular context to favor apoptosis upon genotoxic damage. Mutation of a p53RE controlling a single pro-apoptotic p53 target, *BBC3* (*Puma*), tipped the balance back against apoptosis and allowed the cells to proliferate [89]. In addition to the well-known cell cycle arrest and pro-apoptotic genes, p53 targets also include anti-apoptotic, pro-proliferative genes [6]. One such example is the p53 target gene *KITLG*. The p53RE of the *KITLG* gene has been shown to harbor a single nucleotide polymorphism (SNP) that is associated with reduced p53 binding and *KITLG* expression and increased cancer risk, presumably because the threshold for apoptosis induction is increased in individuals carrying this SNP [90].

Threshold-based mechanisms are clearly not a feature of “smart” p53, but they allow cells to combine a greater number of signals and are less fragile because they are less complex.

### Conclusions and perspective

Over the past years, we have begun to understand the properties that allow p53 to bind to DNA in different chromatin contexts and the co-factors that modulate p53’s ability to transactivate a given locus and control cell fate, although much remains unknown. Yet, when do the well-supported biophysical differences between individual p53 target gene promoters translate into measurable differences in mRNA expression and cell fate? We find that current genomic and transcriptomic data suggest that information content of p53REs is rather low. This could be explained by differences in scales: small differences in p53 binding kinetics and affinity may not be overly relevant in a cell when many other factors are acting on the DNA and p53 has a relatively long time to bind and recruit Pol II. The ability to combine genome-scale assays examining the entirety of sequence space with precise genome editing approaches to validate observations in more native contexts will help resolve these questions. For now, most available evidence suggests that p53 is “dumb” rather than “smart”, trying to transactivate any locus it can bind to (Fig. 1C), instead of decoding information from DNA to selectively activate its targets. We find that it makes sense for a cell to determine its own fate based on simple and robust threshold-based mechanisms rather than on complex and thus fragile p53RE decoding strategies. From an evolutionary perspective, it is difficult to imagine a benefit for shaping and preserving nuances in p53REs to make life-or-death decisions. In the end, the tumor suppressive capacity of p53 is strong no matter whether it is “smart” or “dumb”. However, “dumb” processes might forgive errors such as minor mutations more easily and therefore may be more robust and favored during evolution – at least when it comes to critical decisions such as whether to live or die.
